# Anesthetic management of thoracotomy for massive intrathoracic solitary fibrous tumor of the pleura: a case report

**DOI:** 10.1186/s13019-023-02382-3

**Published:** 2023-10-10

**Authors:** Wang Shen, Yan Li, Feng Liu, Ning Liu, Xiangrui Wang, Zhonghua Ji

**Affiliations:** 1 Department of Anesthesiology, Shanghai East Hospital Affiliated to Tongji University, No.150 Jimo Road, Shanghai, 200120 China; 2 Department of Radiology, Shanghai East Hospital Affiliated to Tongji University, Shanghai, China; 3 Department of Pain, Shanghai East Hospital Affiliated to Tongji University, Shanghai, China

**Keywords:** Solitary fibrous tumor, General anesthesia, Perioperative medicine, Thoracotomy, One-lung ventilation, Intraoperative complications

## Abstract

**Backgrounds:**

Solitary fibrous tumor of the pleura (SFTP) is a rare thoracic tumor and usually asymptomatic. Massive SFTP may affect adjacent organs and tissues including pulmonary vasculature, bronchus and heart. A thoracotomy for massive SFTP is necessary in severe case. Therefore, it is important for anesthesiologists to understand the condition of patients with massive SFTP and develop an appropriate anesthetic management strategy.

**Case summary:**

A 76-year-old woman with massive SFTP presented to our clinical center and was evaluated as requiring thoracotomy. She received multidisciplinary cooperation treatment from the radiology, cardiac, thoracic surgery and anesthetic teams. The perioperative management of anesthesiologists played a crucial role in the great prognosis of this woman.

**Conclusions:**

This case report demonstrates the importance of comprehensive and meticulous perioperative management and provides guidance to the multidisciplinary team on the potential risk and the rational treatment strategy of patients with massive SFTP during the perioperative period.

## Background

Solitary fibrous tumor of the pleura (SFTP) is usually derived from the sub-mesothelial mesenchymal layer, arising from the visceral pleura. SFTP is relatively rare in clinical practice [1]. For example, the incidence of SFTP is approximately 2.8 cases/100 000 people per year [2]. Massive SFTP is defined as a tumor larger than 15 cm in diameter or occupying more than 40% of the hemithorax [3]. Considering the potential complications, such as airway collapse, vascular compression, and hemorrhage, perioperative management of thoracotomy for massive SFTP is a great challenge for anesthesiologists and surgeons [4]. To our knowledge, no reports of comprehensive anesthetic management of thoracotomy for massive SFTP have been published. Herein, we reported our experience of anesthetic management in a thoracotomy for massive intrathoracic SFTP of the left hemithorax with airway and great vessel compression.

### Case presentation

**Fig. 1:**
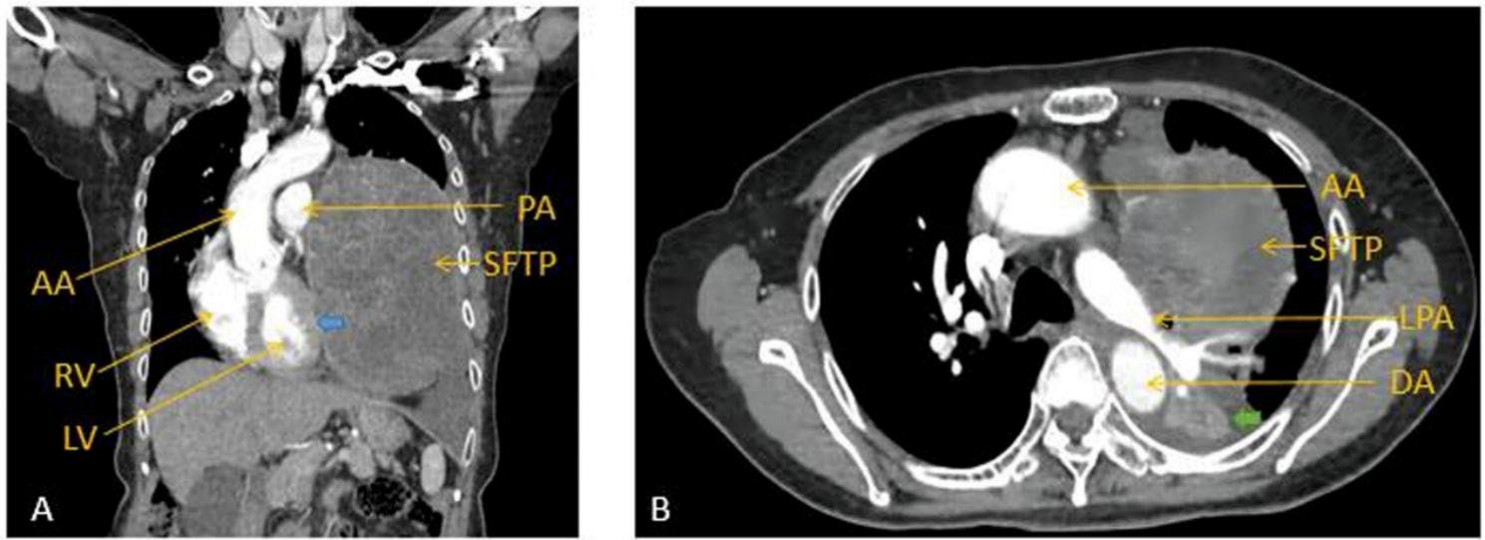
Chest contrast-enhanced computed tomography (CT) demonstrating massive opacification in the left hemithorax. **A**. Coronal image revealed the solitary fibrous tumor of the pleura (SFTP) occupying the majority of left hemithorax. Left ventricle (LV) was compressed by the tumor. The boundary between the pericardium outside the left ventricle and the tumor is not clear (blue arrow). Right ventricle (RV), ascending arota (AA) and pulmonary artery (PA) was pushed to the right. **B**. Axial image showed left bronchial obstruction and left lower lobe atelectasis because of the compression of SFTP (green arrow). Left pulmonary artery (LPA) was compressed severely. Ascending arota (AA) and descending arota (DA) was compressed as well

A 76-year-old woman (62 kg; 164 cm) presented with worsening dyspnea on exertion and moderate asthma. These symptoms began one month before she was admitted to the thoracic surgery department. The chest contrast-enhanced computed tomography (CT) revealed that a large pleural-based mass (craniocaudal diameter of 15.3 cm) with uneven density, vague margin, and inhomogeneous enhancement occupied most of the left hemithorax (Fig. 1A). This space-occupying lesion was medially abutting the left pulmonary artery, ascending aorta, descending aorta, left pericardium, and left ventricle, resulting in the collapse of the left pulmonary vasculature, bronchial obstruction, and lower lobe atelectasis (Fig. 1B), the right shift of mediastinum, pericardium invasion, and left ventricle compression (Fig. 1A). We did not detect abnormalities in multiple laboratory tests except for a slight neutrophil increase. Then, we performed a CT-guided percutaneous transthoracic needle biopsy two days after, which conferred the SFTP diagnosis. Additionally, considering that a huge tumor can considerably affect cardiac structure and coronary artery, we conducted echocardiography, coronary CT angiography (CCTA), and dynamic electrocardiogram to eliminate severe heart diseases such as coronary artery stenosis ventricular arrhythmia. After a comprehensive preoperative evaluation and multidisciplinary discussion, we decided to remove the tumor through thoracotomy.

In the operation room, we applied standard monitoring, including non-invasive blood pressure, electrocardiogram, pulse oximeter, and bispectral index. We established invasive blood pressure monitoring in the right radial artery and inserted the central venous catheter through the right internal jugular vein under ultrasound guidance for intravenous infusion and central venous pressure monitoring. Given the underlying hazard of intraoperative circulatory disturbance, especially cardiac herniation after pericardiectomy, emergency resuscitative strategies, including autologous blood transfusion and cardiopulmonary bypass, were put on standby.

The anesthesia induction was performed in the supine position. First, dexmedetomidine (0.5 µg/mg) was intravenously pumped for 10 min. We ensured that the patient had no dyspnea and precipitous hemodynamic fluctuation under sedation. Next, etomidate 0.3 mg/kg, sufentanil 0.5 µg/kg, and cisatracurium 0.15 mg/kg were intravenously infused successively. Visual laryngoscope-guided double-lumen endobronchial intubation and fiberoptic bronchoscope were used to ensure the proper location of the double-lumen tube in the right main bronchus. We successfully inserted the Robertshaw 32 F catheter at 28 cm from the incisor. We conducted lung auscultation to reconfirm the successful ventilation after lung isolation. The respiratory parameters were adjusted during the operation to maintain end-tidal carbon dioxide partial pressure at 35 to 45 mmHg. Regarding anesthesia maintenance, the target blood concentration of remifentanil was 1 ng/mL. Propofol infusion was given with a target-controlled infusion (TCI) system, and the initial target blood concentration was set at 3.0 µg/mL with adjustments of 1.5–1.7 µg/mL to achieve the expected sedation level by monitoring the BIS value from 45 to 60. The inhalation concentration of sevoflurane was maintained at 1%. The Marsh parameter model was referenced, and the ideal concentrations of propofol and remifentanil were set at 10 mg/mL and 20 µg/mL, respectively. Besides, cisatracurium was intravenously pumped based on the train-of-four (TOF) stimulation value. We used a heated transfusion and insulation blanket to ensure the stability of the patient’s temperature during the surgery.

The surgery was performed in the right lateral position. We removed the whole tumor, the left upper lobe, and part of the pericardium that adhered to the tumor, as well as a portion of the phrenic nerve fused in the tumor and the pericardium. To improve postoperative respiratory function, we conducted diaphragmatic plication after tumor resection. After the resection, we properly performed sputum aspiration and lung inflation to avoid any pulmonary hemorrhage or air leakage. However, when the surgeon used a vacuum extractor to attract the drainage tube placed outside the pericardium after suturing the chest wall incision, the blood pressure sharply dropped to 40/20 mmHg. Meanwhile, the heart rate went up to 180 beats/min and rapidly decreased to 50 beats/min. The anesthesiologist immediately stopped the surgeon from conducting the negative pressure suction. Then, the blood pressure and heart rate swiftly returned to normal.

The blood loss was approximately 300 ml. The blood pressure, heart rate, and oxygen saturation were normal before leaving the operating room. The patient was sent to the intensive care unit (ICU) for postoperative observation and extubated on the first postoperative day. No evidence of residual disease was detected in the postoperative CT. On the seventh postoperative day, the patient was discharged without any complications.

## Discussion

SFTP is rare in clinical practice and characterized by non-specific symptoms, resulting in the unfamiliarity of surgeons and anesthesiologists. Many SFTP patients are occasionally diagnosed with chest radiographs rather than evident clinical symptoms [5]. Larger tumors are prone to be symptomatic because they compress adjacent structures [6]. However, SFTP might be misdiagnosed as pneumonia, hydrothorax, or pulmonary tuberculosis, delaying surgical treatment [7].

Thoracic CT scans can plainly show the location and size of tumors. For massive SFTP, a thoracic CT might show heterogeneity because of hemorrhage, necrosis, or cystic degeneration [8]. Herein, the contrast-enhanced CT revealed nonuniform density, indistinct edge, and heterogeneous enhancement. Moreover, biopsies are beneficial in distinguishing SFTP from other pulmonary malignant tumors. Importantly, thoracic CTs can reveal how the adjacent structures and organs are compressed, helping surgeons and anesthesiologists assess surgery necessity and difficulty. For massive SFTP, it is necessary to perform an open thoracotomy to completely resect the tumor despite a greater risk of bleeding and trauma [9].

To anesthesiologists, massive SFTPs that can threaten the airways, vasculature, and nerves in the thorax and heart bring great challenges to perioperative management [4]. Hence, preoperative evaluation is crucial so that cardiopulmonary function and surgery tolerance can be better acquainted. In the current case, a chief anesthesiologist conducted a preoperative evaluation and medication guidance for the patient in the ward three days before the operation. The respiratory system assessment was comprehensively performed by analyzing the results of chest contrast-enhanced CT, pulmonary function tests, blood gas analysis, and metabolic equivalent. Physical examination, especially auscultation of the lungs, is essential for massive SFTP patients. We did not find aggravated dyspnea or other discomforts in the right lateral position, awake or asleep, and this procedure was instrumental in evaluating intraoperative management safety. The cardiac assessment was also essential in the preoperative evaluation of this patient. Although the series of cardiac examinations did not indicate severe organic heart or coronary artery diseases, as previously mentioned, it was still reasonable to discreetly consider potential cardiovascular damages during surgery. Based on the above information, the chief anesthesiologist identified the feasibility of surgery and anesthesia with high perioperative risk.

Since circulatory instability was predicted, invasive blood pressure monitoring and central venous catheterization were indispensable. Besides, preparing emergency resuscitative measures, including extracorporeal circulation, is vital for an operation that severely affects the respiratory and circulatory systems.

Anesthesia induction is critical during a thoracotomy for massive SFTP. One lung ventilation will considerably burden the ventilated lung during the surgery. The specific process of anesthesia induction should be determined after carefully considering various factors, including pulmonary function, cardiac situation, surgical factors, and the anesthesiologist’s judgment. In our current case, before the infusion of narcotics and muscle relaxants, we tested the patient’s cardiopulmonary tolerance in sedation on the operative position, then performed the administration and intubation. Preoperative thoracic CT can help evaluate the tracheomalacia and trachea carina displacement in massive SFTP patients so that anesthesiologists can select an appropriate lung isolation strategy. A fiberoptic bronchoscope should be applied to ensure the proper location of the double-lumen endobronchial tube. After lung isolation, careful lung auscultation is vital for determining the ventilation effectiveness of the ventilated lung, especially the superior lobe of the right lung.

The selection of drugs for general anesthesia maintenance should be based on the specific situation. Combining intravenous and sevoflurane inhalation anesthesia might be an ideal, stable, and secure strategy in these cases [10]. Additionally, it is advisable to apply a TCI system to control the blood concentration of anesthetics and narcotics [11]. Under BIS monitoring, the depth of anesthesia and the awakening after surgery can be precisely controlled.

Perineural invasion, especially phrenic nerve invasion, also deserves attention during the surgery. Phrenic nerve invasion may seriously damage the respiratory movement on the same side and disadvantage the prognosis of massive SFTP patients. In the case of severe phrenic nerve invasion, it is reasonable to perform diaphragmatic plication to improve postoperative respiratory function [12]. In this case, a part of the phrenic nerve had to be removed due to fusion in the tumor and pericardium, and the surgeon performed diaphragmatic plication accordingly. The patient’s respiratory function recovered smoothly after the operation.

Proper administration and all-round monitoring during surgery can reduce intraoperative risk. In our case, the patient encountered a dangerous emergency at the end of the operation. As mentioned above, violent blood pressure and heart rate fluctuations occurred when the surgeon performed the vacuum aspiration. When intrathoracic negative suctions are performed by the drainage tube outside the pericardium, the heart might herniate through the pericardial incision, known as “heart herniation” [13, 14]. Fortunately, due to the close monitoring, the anesthesiologist immediately stopped the surgeon conducting vacuum aspiration and the blood pressure and heart rate recovered in one minute. However, solutions to severe heart herniation should be explored more. This perioperative crisis requires the anesthesiologist to promptly apply vasoactive drugs to maintain blood pressure and stabilize heart rate. Meanwhile, the key might be that surgeons focus on ending the mediastinal swinging, restoring the heart to its normal position, and lifting the pericardium’s compression on the heart [13, 15].

In conclusion, this case report presents the whole process of the anesthetic management of thoracotomy for a massive SFTP. We showed that massive SFTPs pose a serious threat to the patient’s respiratory and circulatory systems and bring challenges to the perioperative management of anesthesiologists. Anesthesiologists should meticulously and comprehensively understand the patient’s preoperative condition and thoroughly complete the preoperative preparation. Strengthening anesthesia management and monitoring is critical to effectively reduce severe perioperative complications and improve the patient’s prognosis.

## Data Availability

The datasets used and/or analyzed during the current study are available from the corresponding author on reasonable request.
